# Would dogs copy irrelevant actions from their human caregiver?

**DOI:** 10.3758/s13420-018-0336-z

**Published:** 2018-07-06

**Authors:** Ludwig Huber, Natálie Popovová, Sabine Riener, Kaja Salobir, Giulia Cimarelli

**Affiliations:** Clever Dog Lab, Comparative Cognition, Messerli Research Institute, University of Veterinary Medicine Vienna, Medical University of Vienna, University of Vienna, Veterinärplatz 1, 1210 Vienna, Austria

**Keywords:** Overimitation, Companion dogs, Social learning, Attention, Affiliation

## Abstract

Companion dogs learn easily from humans, including human behavior, human communication, and some aspects of the human-made environment. They benefit from having the opportunity to learn from humans and are able to spontaneously synchronize their behavior with that of their caregiver. Here, we tested whether pet dogs would show a special form of observational learning, one that has been considered uniquely human. Indeed, humans show overimitation, the faithful copying of causally irrelevant actions, but great apes do not. Because in humans this peculiar form of imitation is strongly motivated by social factors, such as affiliation or conformity, we hypothesized that domesticated and enculturated dogs are more likely than apes are to copy such actions, especially if shown by their affiliated caregiver. Indeed, half of the dogs replicated a causally irrelevant action that was demonstrated by their caregiver, and about the same number did this whether they saw only this action being demonstrated or being demonstrated before or after a causally relevant, functional action. The demonstration of a causally relevant action, one that is immediately followed by access to food, thus does not inhibit the copying of an action that is spatially separated and functionally opaque. Given that the copying frequency in this study was low overall, these results suggest evidence for overimitation in dogs, which might challenge the human uniqueness of this type of social learning.

**Electronic supplementary material:**

The online version of this article (10.3758/s13420-018-0336-z) contains supplementary material, which is available to authorized users.

Learning by observation, or often simply social learning, is an important and enduring research topic in cognitive biology. It usually refers to learning that is influenced by observation of, or interaction with, another animal or its products. Textbooks describe many examples of the adaptive modification of behavior through learning from others, ranging from food selection and predator avoidance to learning songs, routes, and motor skills. In social species, this kind of learning often results in synchrony in the performance of established behavior (sometimes linked to conformity) and the transmission of new behavior patterns throughout a group. These effects are mostly (though not necessarily and not always) beneficial to the observer, either immediately or long term, and are therefore assumed to outweigh potential costs in terms of vigilance, attention, memory, or special learning mechanisms.

Despite its role in the ontogeny of adaptive behavior, as well as its importance in human culture, sometimes the result of learning by observing adults may be nonfunctional or even maladaptive. Humans often imitate in less efficient manners, including actions that appear causally irrelevant, like first tapping the side of the jar with a feather and then unscrewing the lid in order to retrieve a dinosaur from a plastic jar (Lyons, Young, & Keil, 2007). This phenomenon, called “overimitation,” appears to be uniquely human (Horner & Whiten, 2005; Legare & Nielsen, 2015; Lyons et al., 2007; McGuigan, Whiten, Flynn, & Horner, 2007; Whiten, McGuigan, Marshall-Pescini, & Hopper, 2009).

Several theories have been proposed as to why overimitation occurs, although so far none have satisfactorily explained all the factors. One possibility is that children believe that the noncausal actions that are intentionally shown by adults are somehow necessary to achieve the final goal (Lyons et al., 2007). They implicitly revise their causal understanding of the manipulated object, which allows them to rapidly calibrate their causal beliefs about even the most opaque physical systems, such as switching on the light. However, children are principally able to differentiate between necessary and irrelevant actions, suggesting that they do not believe the noncausal actions truly serve a mechanistic purpose (Kenward, Karlsson, & Persson, 2011). More generally, in causally opaque systems, any action performed before achieving the goal is likely to be inferred as causally necessary—this is not overimitation, but ordinary causal learning.

Therefore, some researchers suggest that overimitation occurs because children interpret the demonstrated actions as social or cultural conventions or norms that should be copied. Indeed, the children claim to be unsure as to why a causally unnecessary action has to be performed, but insist that it has to be done (Kenward et al., 2011). Finally, several developmental psychologists propose that children overimitate the actions of adult demonstrators in order to promote affiliation with the demonstrator (Meltzoff, 2007; Nielsen, 2006; Over & Carpenter, 2013).

The human uniqueness of overimitation—the faithful copying at the expense of effectiveness—has been claimed since its first comparison with chimpanzees (Horner & Whiten, 2005). In a recent study, not a single bonobo copied any of the causally irrelevant actions, but the great majority of children did (Clay & Tennie, 2017). The authors argue that this provides qualitative and ecologically valid evidence of the distinctive nature of the human cultural capacity as compared with great apes. However, overimitation appears first in children around 3 years of age (McGuigan et al., 2007), and it increases with age over childhood (McGuigan, Makinson, & Whiten, 2011; Taneguchi & Sanefuji, 2017); thus, it seems that it is a result of early enculturation rather than its cause. In line with this assumption is the fact that the best evidence for imitation in great apes comes from enculturated apes, those that have been raised by humans (Carrasco, Posada, & Colell, 2009) or have received extensive training (the so-called do-as-I-do studies; Call, 2001; Custance, Whiten, & Bard, 1995; Hayes & Hayes, 1952).

Companion dogs are special with regard to enculturation and the establishment of a close bond with humans. Therefore, it is reasonable to explore how dogs would copy human actions. Earlier research has shown that dogs benefit from having the opportunity to learn from humans (Pongrácz et al., 2001; Pongrácz, Miklósi, Kubinyi, Topál, & Csányi, 2003). Even more, dogs are able to synchronize their behavior with that of their owners without any reinforcement by being able to anticipate the owner’s action (Duranton, Bedossa, & Gaunet, 2017; Kubinyi, Topál, Miklósi, & Csányi, 2003) and, like children, they interpret a test situation often as being a form of communication or social game (Soproni, Miklósi, Topál, & Csányi, 2001).

Dogs learn from humans especially well if trained in the so-called do-as-I-do paradigm. They learn to copy not only a few trained actions but also novel actions and action sequences (Fugazza & Miklósi, 2014; Fugazza, Pogány, & Miklósi, 2016a, 2016b; Huber et al., 2009; Topál, Byrne, Miklósi, & Csányi, 2006). Their actions seem to be goal-directed, and shortcuts reveal that they are often driven by efficiency (Huber et al., 2009). However, like apes (e.g., Call, 2001; Myowa-Yamakoshi & Matsuzawa, 1999), they show similar tendencies of perseveration, as in novel situations they fall back into the attractors of training actions. And finally, superior performance with (transitive) object manipulations in comparison with (intransitive) body-oriented movements like gestures (e.g., Tennie, Call, & Tomasello, 2009) is not only congruent with the findings from great apes but also with those of children with autism (Heimann, Ullstadius, Dahlgren, & Gillberg, 1992).

One test in the study by Huber et al. (2009) was especially informative with regard to the question of overimitation. It tested if the dog, a female adult Weimaraner named Joy, would copy “blindly” or would try to make sense of the action and then recreate the most effective or “rational” solution. It required the dog to copy actions for which the target object was not (or no longer) present (so-called vacuum actions). For instance, the human demonstrator (the caregiver) “jumped over nothing,” “imitated drinking (on all fours on the ground) from nothing,” and “put a ball into nothing.” Without exception, the dog failed to replicate the shown action faithfully but responded by performing an action that was in context or functionally similar. In the case of jumping, she jumped over the “real”’ hurdle, which was standing nearby.

Although such “rationalizing” copying behavior in dogs was also shown in a task that replicated “rational imitation” in preverbal children (Gergely, Bekkering, & Kiraly, 2002), by imitating a conspecific selectively based on an inference about the necessity or efficiency of the matching response (Range, Virányi, & Huber, 2007), it is not clear why Joy resisted copying “irrational” or object-missing actions. Prima facie, this result may speak against overimitation in dogs.

The answer to the question is blurred by the ambiguity of results of other studies in which dogs were asked to learn from human demonstrations. On the one hand, not only in spatial learning tasks (the detour problem; Pongrácz et al., 2001) but also in object manipulation tasks (Kubinyi et al., 2003; Pongrácz, Bánhegyi, & Miklósi, 2012), dogs showed demonstrator-matching behavior after observing a human. That humans are efficient demonstrators was also shown in Miller, Rayburn-Reeves, and Zentall (2009), who found that dogs would not only manipulate the same object—a sliding door blocking access to food—but they would even match the direction of the door push (leftward or rightward) demonstrated by a human (see also, Kis, Huber, & Wilkinson, 2015; Veit, Wondrak, & Huber, 2017). This study has thus provided some evidence for imitative learning from humans, not only stimulus enhancement.

In contrast to this positive evidence for imitative learning in dogs, Mersman and colleagues (2011) failed to report a facilitating effect of seeing a skillful demonstrator solving an instrumental task (successfully pulling a towel to reach food). There was no evidence that subjects copied any of the actions of the demonstrator—for instance, by using the same body part (paw or mouth) as the demonstrator.

In humans, the faithful copying of causally irrelevant actions (overimitation) is strongly motivated by social factors, such as to affiliate with or “be like the other” (Keupp, Behne, & Rakoczy, 2013; Nielsen, 2006). Therefore, it is possible, that dogs would copy such actions if shown by the affiliated caregiver. Indeed, dogs attended more to familiar humans with whom they also had a close relationship (Horn, Range, & Huber, 2013). This might be further facilitated by ostensive cues, for instance, eye contact and dog-directed speech, as has been used by Kubinyi et al. (2003). A couple of studies have shown that dogs’ sensitivity to ostensive cues parallels that of human children (Topál, Gergely, Erdőhegyi, Csibra, & Miklósi, 2009). For example, like preverbal infants, dogs follow a human’s gaze only if it is preceded by ostensive cues (Téglás, Gergely, Kupán, Miklósi, & Topál, 2012; Wallis et al., 2015), and they commit perseverative errors following ostensive communication (e.g., Péter et al., 2015; Topál et al., 2009).

Only recently have dogs (and dingoes) been tested to solve a puzzle after watching an ostensive demonstrator who showed two actions on a puzzle box, of which only one was causal for its opening (Johnston, Holden, & Santos, 2017). The great majority of dogs (75%), and a smaller number of dingoes (54%), replicated both actions on test (Trial 1). This copying of both the relevant and the irrelevant action was less faithful than in children, as shown by a smaller proportion of subjects, and it was less enduring across further trials. Still, even after four trials, about half of the subjects performed the irrelevant action, the manipulation of a lever. Importantly, in all these respects the dogs differ largely from both chimpanzees (Horner & Whiten, 2005) and bonobos (Clay & Tennie, 2017), which did not show the copying of the irrelevant action at all.

From a theoretical point of view, it is, of course, disputable whether the high number of dogs showing the irrelevant action in the first trial is evidence in favor of overimitation—as most studies so far implied—or whether the decrease in lever use across trials is evidence against overimitation, as Johnston et al. (2017) argued. The answer to this question depends very much on the definition of this phenomenon, and the explanation for its occurrence. In humans, it also depends on the experimental circumstances, as the rate of overimitation decreased significantly when the participants faced time pressure (Flynn & Smith, 2012). Still, the main issue is the copying of what has been termed “causally irrelevant actions.” The weakness of this crucial feature of the phenomenon is its anthropocentric bias. From the experimenter’s point of view, it is easy to design a problem-solving task by including one causally relevant and one causally irrelevant action—that is, one that immediately provides access to the treat (a toy or a piece of food) and one that does not. But from the observer’s point of view, this might not always be obvious. Simply making the food box transparent (Horner & Whiten, 2005) may not be enough. It may be sufficient for technically skillful chimpanzees to understand that a stabbing action in the upper part of the box has no effect onto the food release in the lower part of the box, especially if there is a platform that physically separates the two parts. But for dogs the causal transparency is not obvious. How should a dog know from a meter distance that a protruding lever has no effect on the releasing/opening mechanism of the box? Only because it has no immediate effect?

Here, we devised a different experimental design to test outcome-insensitive or “blind” copying in dogs. Like in most studies on overimitation, we confronted the dogs with a typical overimitation action sequence, that is, the demonstration of a causally superfluous action A, an effect-relevant action B, and effect E. To increase the causal transparency of the “irrelevant” action, it was spatially separated from the food box. Children did not overimitate in such a condition of physical/spatial separation of cause and (potential) effect (Lyons et al. 2007; Experiment 2b; but see Keupp et al., 2013, and Taniguchi & Sanefuji, 2017, for different results). In addition, the irrelevant action was functionally separated from the food extraction action (pushing a sliding door to the side to provide access to the food) by showing a kind of intransitive or outcome-insensitive action (touching color blobs on a sheet of paper). Furthermore, we asked the human caregiver, not an unfamiliar experimenter, to show the actions after ostensively addressing the dog.

We expected that some dogs would spontaneously copy both actions because it is understood as a social game and a way to please the human partner. However, we did not expect all subjects performing both actions. On the one hand, we expected that dogs that had received special education, like target training (e.g., touching an object with the nose) at home or touch-screen training in the Clever Dog Lab, would overimitate. Those enculturated dogs have learned to follow the commands and demonstrations of humans, especially their human caregiver. On the other hand, observational learning requires a high degree of attentiveness during observation and a good memory to retrieve both actions when subsequently being tested. For these reasons, we asked the caregivers to tell us the nature and the amount of training their dogs received before the onset of the experiment, and applied an attention test. This test was administered shortly before the observation experiment and involved six object-permanence trials including an A-not-B trial. Using a between-subjects design (four groups), we controlled for primacy and recency effects by manipulating the position of the irrelevant action in the action sequence and for the likelihood of the copying of the two kinds of action when showing them alone or together.

## Method

### Ethics statement

All procedures applied in this study were discussed and approved by the institutional ethics committee of the University of Veterinary Medicine Vienna, in accordance with good scientific practice guidelines and national legislation (Ref. 03/08/2017). All dog owners volunteered to participate with their dogs in this study and gave written consent. The experimental procedure was purely noninvasive, and handling of the dogs was always in a positive and pleasant manner.

### Subjects

Seventy-two dogs (34 males, 38 females) of various breeds (except very small breeds) ranging in age from 18 months to 13 years (*M*_age_ = 5.3 years) were recruited to participate in the study. All recruited dogs were kept as family pets and voluntarily brought to the Clever Dog Lab Vienna by their owners. The owners gave written consent and provided the necessary information about their dogs (keeping conditions, training history, earlier participation in the Clever Dog Lab, etc.). Twelve dogs were excluded due to lack of motivation or because of technical problems with the video recordings. The remaining 60 dogs were divided into four equally sized (each *N* = 15) groups with counterbalanced sex, age, training history, and breed composition. The individual characteristics of those dogs, including their training history (such as target training at home or touch-screen or eye-tracking training in the Clever Dog Lab) are provided in Table S1 of the Supplementary Material.

### Experimental setup

**Fig. 1.**
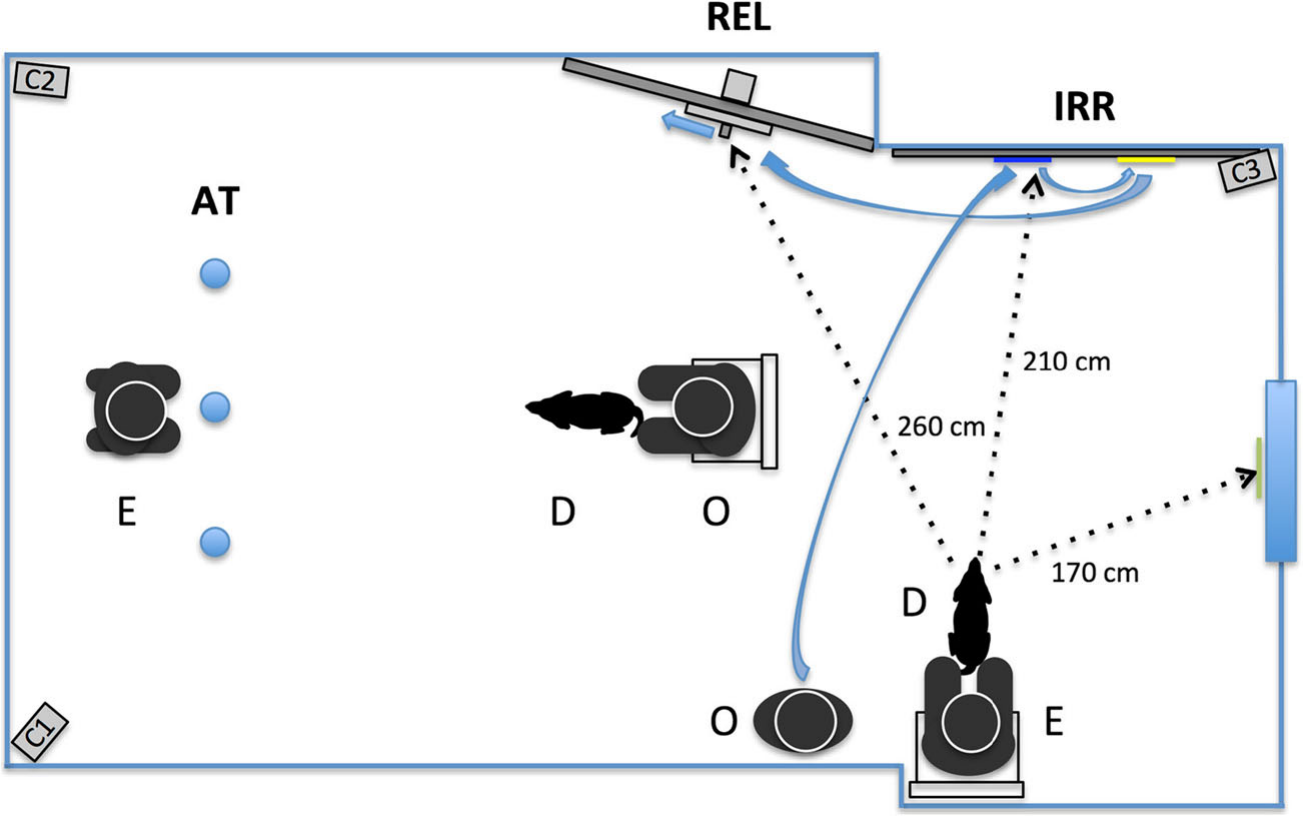
Experimental setup during attention test and imitation test. AT = attention test; REL = causally relevant action; IRR = causally irrelevant action; D = dog; E = experimenter; O = owner; C1, C2, C3 = video cameras. Solid arrows show the movement trajectory of the owner when demonstrating the IRR+REL action sequence. Dotted arrows show the distances between the dog waiting at the starting position and the objects in the imitation test (see Method section)

The dogs were tested in a midsized room (6.0 × 3.3 m) of the Clever Dog Lab Vienna. This room was equipped with three video surveillance cameras to record the experimental trials. The cameras were fixed to the walls at approximately a 2 m height and positioned with the aim to film the details of the dog’s performances during the tests (see Fig. 1).

For the attention test, three opaque, blue-painted cups (10.5-cm high, 8.5-cm diameter) were positioned on the floor in a straight line. The distance between two adjacent positions, from center to center, was 65 cm.

**Fig. 2.**
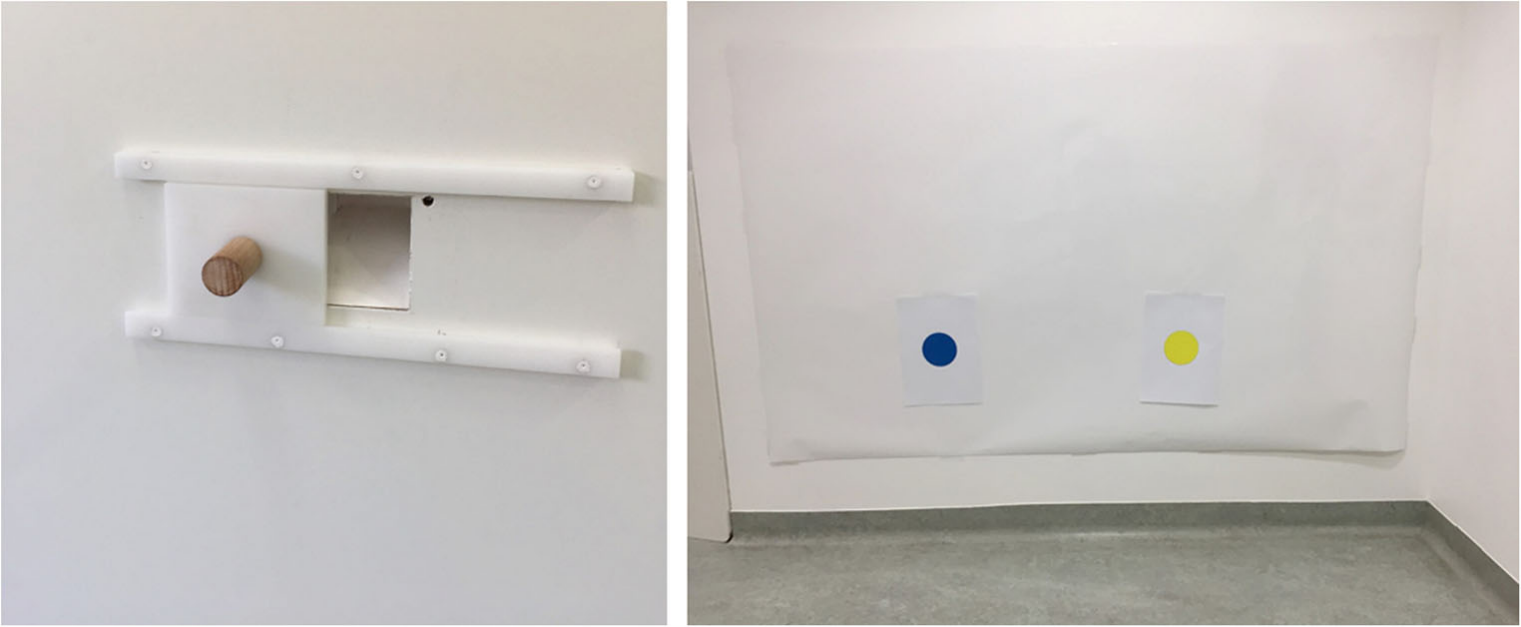
Two targets of the imitation test. Left: Sliding door affording the causally relevant action (opening). Right: Paper wall with color dots affording causally irrelevant actions (nose touching)

For the social learning test, one of the long side walls was modified by installing a white wooden plate (150 × 100 cm) to cover a corner in the wall. At the central position of this wall and 50 cm above the floor, we made a rectangular cutout (6 × 7 cm) and placed a food receptacle with a skewed floor behind it. This hole was completely covered by a white, wooden, rectangular sliding door (10 × 9 cm) that could be moved within two 30-cm long table tracks (one above, one below) an equal distance (9 cm) to the left or right of the hole. To facilitate moving, we fixed a brown wooden handle (4-cm long, 2-cm diameter) to the sliding door (see Fig. 2). The food box could be filled with a piece of sausage. If the subjects were allergic to the ingredients of the treats, we used alternative, same-sized treats provided by their owners.

At the same wall, but 130 cm away from the sliding door, we mounted a white, laminated poster sheet (172 × 106 cm). On this paper wall we glued two white, A4-sized (30 × 20 cm) sheets of paper (standard reprographic paper) with centrally located, printed color dots (9 cm in diagonal; one blue, the other yellow; see Fig. 2). The distance of the color dots (center) from the floor was the same as for the sliding door (50 cm); the distance between the centers of the color dots was 60 cm. To prevent any scent transmission, every dog got new sheets of A4 paper with color markers, glued on the wall, which was cleaned before each test. Nevertheless, to ensure that the dogs did not use their sense of smell to decide which part of the room to approach and explore after the observation of the demonstrator’s actions, another sheet of white paper could be glued to the center of the entrance door at the height of 50 cm. It was touched by the owner after the attention test and shortly before the observation trial and thus served as a control for any scent cue left by the owner.

On the opposite side of the paper wall, a chair was provided for the experimenter to sit in while holding the dog on a short leash during the demonstration phase. From this position the dogs could see the sliding door at a distance of approximately 260 cm, the colored markers at a distance of approximately 210 cm, and the scent-control sheet on the door at a distance of about 170 cm (see Fig. 1).

### Design and procedure

Before the experiment, we instructed the owners verbally and with a video example of what to do and what not to do during the testing of the dog. We purposefully kept them uninformed about the aim and hypotheses of the study and what we predicted the dogs would do. We asked them to behave as naturally as possible in front of their dog, thus providing a safe haven and secure base for them (Horn, Virányi, Miklósi, Huber, & Range, 2012).

### Attention test

This pretest aimed at assessing the attention level of each animal prior to the actual test. This followed the logic that only attentive and motivated dogs would observe the demonstrated actions carefully (Range, Horn, Bugnyar, Gajdon, & Huber, 2009). The test consisted of six object hide-and-search trials with three hiding locations (cups). Each trial started with the experimenter (E) kneeling centrally behind the cups (see Fig. 1). She showed a piece of food by raising her hand, waited until the dog looked at her, and then bent down and visibly hid the treat by opening the lid of the target cup, putting the treat inside, and closing the lid. The sequence of hiding locations was always the same: the first three times was the middle cup, then the left cup (which we considered as M-to-L change), then the right cup, and finally again the middle cup. The first four trials aimed at testing the dogs’ tendency of committing the so-called A-not-B error (Kis et al., 2012; Piaget, 1954). The owner was sitting on a chair in the center of the testing room and kept the dog on a long leash (see Fig. 1). The distance between the dog’s head and the three cups was 180 cm (using a marker on the floor). After E finished hiding the treat, the owner let the dog go (still keeping the dog loosely on the leash), and the dog searched for the hidden treat. During a trial, if the animal chose the target container first (approach and touch), the experimenter opened the cup and allowed the dog to eat the piece of sausage. If the animal chose a nontarget container first, the experimenter quickly took away the two other cups and the owner called the dog back. The attention tests lasted approximately 3 minutes. In the Supplementary Material, Movie S1 shows example trials from the attention test.

### Imitation test

The imitation test followed after a break of approximately 5 minutes, during which the dog remained outside the experimental room. The experimenter recapitulated the sequence of the demonstrator’s actions and asked the demonstrator to do a “trial run” and then to wipe his or her nose three times over a white sheet of paper that was then glued to the door as a scent control. The dog was then brought back into the room, and the imitation test was started.

Four groups of dogs (each *N* = 15) saw different actions demonstrated by their owners. To assess the baseline probability of copying the actions, we tested two groups that saw only one of the two actions demonstrated; group REL saw the causally relevant action (i.e., the moving of the sliding door); group IRR saw the irrelevant action demonstrated (i.e., the touching of the color dots). The two other groups saw both actions demonstrated—one group (IRR+REL) in the usual overimitation sequence (i.e., first the causally irrelevant action followed immediately by the causally relevant action and the other (group REL+IRR) in the reverse order. In the Supplementary Material, Movie S2 shows an example of a human demonstration of the IRR+REL condition.

The test started with the experimenter bringing the dog to the observer position (see Fig. 1), sitting down on the chair behind it, and holding the dog on a short leash. The owner stood to the left to the experimenter and waited until the dog was sitting quietly. The owner then gave the dog a treat to gain the dogs’ attention and started the demonstration. An important aspect of the demonstration was the attempt to show the actions in a dog-like manner: The dogs had to copy the actions faithfully—by using the same body part—and thus showing true imitation (Huber et al., 2009; Voelkl & Huber, 2000, 2007), which is a prerequisite of overimitation. That is, rather than moving the sliding door with their hands, which is not possible for dogs, the dog owners were asked to do it with their noses. Also, they touched the color dots with their noses. Both actions had been performed in a dog-like position (i.e., on hands and knees). When required to show both actions in a sequence (for groups IRR+REL and REL+IRR), the owners were asked to do the actions in immediate succession. The one-action demonstrations lasted approximately 10–20 s; the demonstration of both actions lasted approximately 30–50 s.

In all four groups, the dogs observed the human’s demonstration from behind. From their point of view, the dogs saw the owner approaching the paper wall from the left side and the sliding door from the right side with an angle of approximately 45 degrees to the wall.

The classical overimitation demonstration has two parts: the irrelevant action followed by the relevant action (Horner & Whiten, 2005; Johnston et al., 2017). Only one of our groups (*N* = 15) was tested in this manner (IRR+REL; see Fig. 1). The demonstration of the causally irrelevant action consisted of touching the two color dots on the paper wall. The owner moved toward the paper wall, got down on her hands and knees, and touched first the blue and then the yellow dot with her nose. Then, the owner walked ca. 1.5 m to the wooden wall with the food box, got down on her hands and knees, and opened the food box by pushing the handle of the sliding door with her nose leftward by 9 cm. She then took the piece of sausage and showed it to the dog. Afterward, she turned again to the sliding door, obscuring it with her body, and put the treat, out of the dog’s view, back into the food chamber. Immediately after returning to the starting position next to the demonstrator, the dog was released from the leash by the experimenter and the test started.

Everything else being equal, the dogs in the REL+IRR group saw the same demonstration but in reverse order. The aim of this group was to control for sequence effects such as primacy or recency effects (Murdock, 1962). It also tested for possible effects of one action on the other. Importantly, rather than having both actions performed on the same apparatus, as in most overimitation studies (e.g., Horner & Whiten, 2005; Johnston et al., 2017), the dogs’ owner demonstrated actions on different locations and objects. Moreover, the causal disconnection in this study was emphasized in several ways. First, touching the color dots was completely ineffective, causing no change in the environment. Secondly, the distance between the targets of the two actions was large (130 cm). Thirdly, the temporal connection between the two actions was loose (several seconds). And finally, the two action locations were separated by salient movements of the demonstrator (standing up, walking, kneeling down).

The aim of the two single action groups was to assess the baseline probability of the two actions without being influenced by memory effects or influences from the other action shown immediately before or afterward.

In contrast to Johnston et al. (2017), the dogs were tested in the presence of the owner and the experimenter, to avoid the dog becoming stressed by the disappearance of the humans and remaining alone in the room. The dog was released and allowed to explore the room and possibly also copy the shown actions. The owner was instructed to remain passive and to refrain from interacting or communicating with the dog. Three digital video cameras that were connected to a video-recording station outside the test room were used to videotape the tests. The positioning of the cameras allowed the recording of the most relevant details, namely, the attention and performance of the dogs in the attention test (C1; see Fig. 1), as well as the manipulation of the sliding door (C2) and the touching of the color dots (C3) in the imitation test.

### Data analysis

The three video streams of the Clever Dog Lab’s video-recording system had been merged and then used for coding with the behavior coding software Solomon coder beta (© 2015 by András Péter). A correct response in the attention test was defined as touching the hiding cup first. For the social learning test, we coded for the first minute of the test (starting with the dog being released by the experimenter) the time (in seconds) the dogs spent gazing toward their owners during the demonstration, sniffing the scent control sheet (yes or no), sniffing the dots (yes or no), sniffing the sliding door (yes or no), touching the blue dot (yes or no), touching the yellow dot (yes or no), the order of touching the dots (blue > yellow or yellow > blue), pushing the sliding door leftward (yes or no), pushing the sliding door rightward (yes or no), and the order of the actions (door > paper or paper > door).

#### Attention test

The total number of correct responses was compared against chance level using a one-sample *t* test, while the number of dogs correctly choosing the M-to-L change were compared against chance level using a binomial test. For both tests the chance level was set at 33%.

#### Imitation test

To investigate whether the action shown during the demonstration positively affected the actions shown during the test, we used a Fisher’s exact test (one sided) to compare the distribution of dogs across groups: opening the sliding door (IRR vs. other three groups); touching at least one color dot (REL vs. other three groups); touching both color dots (REL vs. other three groups); touching at least one color dot and opening the sliding door; as well as touching both color dots and opening the sliding door (only one action demonstrated [REL or IRR] vs. both actions demonstrated [IRR+REL or REL+IRR]). Moreover, we used a Pearson chi-square test to compare the likelihood of showing a specific behavior during the imitation test across the four different groups.

In order to investigate whether a higher performance during the attention test was associated with a higher likelihood of showing the demonstrated behaviors dogs showed during the imitation test, we ran a Mann–Whitney *U* test to compare the number of correct responses during the attention test between dogs that showed and did not show a specific behavior, and a Fisher’s exact test (one sided) to compare the distribution of dogs correctly choosing during the M-to-L change between dogs that showed and did not show a specific behavior.

Moreover, we investigated whether the attention showed during the demonstration affected the distribution of dogs showing a specific behavior during the main test. In particular, we compared the percentage of time that dogs spent gazing toward their owners during the demonstration across dogs that showed or did not show a specific behavior using Mann–Whitney *U* tests.

Finally, we investigated whether training (see Subjects) affected dogs’ performance during the main test by conducting a Fisher’s exact test (one sided) comparing the distribution of dogs showing a specific behavior between trained and nontrained dogs. All tests were conducted using SPSS Version 24 (SPSS Inc., Chicago, IL, USA).

### Interobserver reliability

Three experimenters coded the videos and one additional coder, unaware of the aim of the study and the hypotheses, coded a random subsample (25%) of videos. This naïve coder could only see the behavior of the dogs; the demonstration of the owner was digitally removed. Interobserver reliability for the three main coders was very high (for all variables, Cronbach’s α > .98), the comparison with the naïve coders provided good interrater agreement (for all variables, α > .79).

## Results

### Attention test

Dogs performed above chance in the attention test (mean ± *SD* = 55% ± 27.84%), one-sample *t* test, *t*(59) = 6.03, *p* < .001, but did not differ from the chance level in the M-to-L change (binomial test, *p* = .07).

### Imitation test

From all 60 dogs tested, 39 sniffed at the sliding door and 28 sniffed at the dots (see Table 1). While the first did not show a statistically significant group difference (chi-square test, *p* = .11), the second revealed a significant group difference (chi-square test, *p* = .03). Post hoc tests revealed that the two-action groups exhibited more sniffing at the dots than the REL group. There was also a significant group difference for the sniffing of both the door and the dots (chi-square test, *p* = .04). While only two of the 15 dogs (13.33%) that observed only the door opening sniffed the dots, 26 of the 45 dogs (57.78%) that observed the owner touching the dots sniffed at them.

**Table 1:** Dog’s behavior in the imitation test

Dog’s performance	*N*	IRR+REL	REL+IRR	IRR	REL	Stat
Sniffing door	39	12	10	6	11	>.1
Sniffing dot(s)	28	9	9	8	2	.03
Sniffing door + dot(s)	23	9	7	4	2	.04
Touching at least one dot	24	7	7	8	2	>.1
Opening door anyhow	15	5	6	0	4	.06
Touching both dots anyhow	11	4	4	3	0	>.1
Opening door correctly	11	5	3	0	3	>.1
Touching dots correctly	3	2	0	1	0	>.1
Touching one dot and opening door anyhow	8	3	3	0	2	>.1
Touching one dot and opening door correctly	5	3	1	0	1	>.1
Touching both dots anyhow and opening door anyhow	4	3	1	0	0	.09
Touching both dots anyhow and opening door correctly	3	3	0	0	0	.02
Touching both dots correctly and opening door anyhow	2	2	0	0	0	>.1
Touching both dots correctly and opening door correctly	2	2	0	0	0	>.1

*Note.* Various behaviors of dogs in the imitation test, ordered according to level of copying precision (from low to high). *N* is the total number of dogs, and the next four columns the number of dogs in the four experimental groups (see Method section) showing the behavior. Stat gives the *p* value of the Pearson chi-square test comparing the distribution of behaviors across the four groups to evaluate how likely it is that the observed difference between the groups arose by chance

Of course, sniffing is not copying the action, but the fact that no dog touched the scent control dot (0/60) would support an account in terms of an effect of observation. Copying the causally irrelevant action would require touching the dots. Approximately half of the dogs (22 out of 45; 48.9%) that observed the owner touching the dots did so with at least one dot (see Table 1). The dogs that did not observe dot touching (group REL) were less likely to touch at least one dot (2/15 REL vs. 22/45 other three groups; Fisher’s exact test, *p* = .01).

Faithful copying of the irrelevant action would require touching both dots. This was shown by 11 dogs, more or less equally distributed across the three groups that observed it, but by none of the group that observed only the door opening (REL; Fisher’s exact test, *p* = .03). Still, the precise copying of the irrelevant action would require touching both dots in the observed sequence (first the blue one, then the yellow one), which would indicate sequence imitation. However, only three dogs performed such precise matching, two of the overimitation group and one of the IRR group (see Table 1).

Concerning the copying of the causally relevant action, 15 dogs pushed the door and got the treat. These dogs observed both actions (11) or only the door-opening action (4). Not surprisingly, none of the dogs of group IRR opened the door, despite the fact that the food box was filled with a smelly piece of sausage. Again, observing the action had a significant effect on the matching performance in the test (0/15 IRR vs. 15/45 other three groups; Fisher’s exact test, *p* = .006). Moreover, dogs were less likely to do both touching the dots and opening the door if they had seen only one action (no matter which) during the demonstration (0/30 REL and IRR vs. 4/30 IRR+REL and REL+IRR; Fisher’s exact test, *p* = .06). However, dogs did not seem to differ in regard to touching at least one dot and opening the door (2/30 REL and IRR vs. 6/30 IRR+REL and REL+IRR; Fisher’s exact test, *p* = .13).

Only two dogs faithfully copied both actions, the touching of both dots in the right order and the opening of the door in the right direction, both being in the overimitation (IRR+REL) group (chi-square test, *p* = .10). In the Supplementary Material, Movie S3 shows an example of a dog performing both actions in the IRR+REL condition.

### Effect of attention

The performance during the attention test did not affect how dogs performed during the main test (Fisher’s exact test, all *p*s > .05). However, dogs that touched the dots at least once looked longer at their owners during the demonstration (Mann–Whitney *U* test, *U* = 659.50, *p* = .001). None of the other behaviors were associated with the attention shown during the demonstration (Mann–Whitney *U* test, all *p*s > .05).

### Effect of training

Trained dogs were more likely to open the lid (Fisher’s exact test, *p* = .005), open the lid in the right way (Fisher’s exact test, *p* = .02), touch both dots (Fisher’s exact test, *p* = .02), touch both dots and open the lid (Fisher’s exact test, *p* = .049), touch one dot and open the lid (Fisher’s exact test, *p* = .002), touch one dot and open the lid in the right way (Fisher’s exact test, *p* = .02), and were more likely to touch at least one dot (Fisher’s exact test, *p* = .06). Both dogs that touched the dots in the right order and opened the lid in the right way were trained, but due to the low number of individuals, the statistical test did not reach significance (Fisher’s exact test, *p* = .23).

## Discussion

This study revealed that about 50% of companion dogs copied a causally irrelevant or functionally unnecessary action that was ostensively demonstrated by their human caregivers. About the same number of dogs did this whether they saw only this action or an action sequence together with a causally relevant, goal-directed, or functional action. This means that the demonstration of a causally relevant action, one that is immediately followed by access to food, does not inhibit the copying of an action that is spatially separated and functionally opaque. Furthermore, the perfectly matching action sequence—touching the dots in the right order and then moving the lid in the right direction—was shown only by dogs that saw exactly this action sequence demonstrated. And at a slightly lower level of precision—touching both dots, regardless in which order, and then moving the lid in whatever direction—was shown only by dogs that saw both actions demonstrated (regardless in which order). Given that overall the copying frequency in this study was low –only four of 15 dogs copied the relevant, door-opening action after seeing it demonstrated alone—these results suggest evidence for overimitation in dogs.

Recently, Johnston et al. (2017) interpreted the decreasing frequency of performing the irrelevant action across trials as evidence against overimitation. This is surprising, because the great majority of dogs in this study have copied the irrelevant action in the first trial. One could, of course, follow the authors’ argument that the decrease, however strong, is an indication that the dogs learned that the irrelevant action had no function and is therefore being abandoned. The researchers reported that after three times experiencing that the first, irrelevant action is not immediately followed by access to food, half of the sample of dogs still performed this action, which was about the same number of dogs performing the relevant action.

The comparison of our data, as well as those from Johnston et al. (2017), with those from humans (Lyons et al., 2007) and great apes (Clay & Tennie, 2017; Horner & Whiten, 2005), reveals an intermediate level of overimitation in companion dogs. These animals are less prone to copy the irrelevant action than humans are, but still some do, while none of all tested great apes did. This provokes the question of whether this pattern of results needs an explanation in terms of the underlying observational learning mechanisms or in terms of the relationship between demonstrator and observer.

Concerning the mechanism that dogs have used to copy the relevant or irrelevant actions, for most subjects it is sufficient to assume stimulus and local enhancement (Mersmann, Tomasello, Call, Kaminski, & Taborsky, 2011). Only for those few dogs that copied the observed actions in all details, one could assume true imitation (Voelkl & Huber, 2000, 2007). However, without a two-action test or bidirectional control procedure (Miller et al., 2009), any conclusion about imitative learning must remain tentative. It is interesting that in the REL condition, in which dogs were only presented the sliding of the door, only three out of 15 dogs (20%) copied the action precisely (i.e., in the right direction). This contrasts with the results of Miller et al. (2009), in which nine of 12 dogs (75%) in the first trial pushed the screen in the same direction that they saw the screen-push demonstrated by a human. There are several possible reasons for this discrepancy. In Miller et al., the screen was 35.5 cm high × 30.5 cm wide and could be moved 16.5 cm to the left or right of the hole, whereas in our study the screen was much smaller (10 × 9 cm) and the respective movement much shorter (9 cm). Furthermore, in Miller et al., the dogs were placed at a distance of about 90 cm from the screen and at a 45° angle to the center panel. They could see the screen while it was being pushed by the human’s hand, both from the left and the right side. In our study, dogs could observe the door pushing from a distance of 260 cm and with the human caregiver partly blocking the view to the screen with her head (we asked them to open the screen with the nose). But most importantly, the dogs in Miller et al. had 12 demonstrations of the human opening the screen with the hand, six from a position to the right of the screen and six from a position to the left of the screen. Our dogs had only one demonstration before being tested.

Overimitation in humans has been explained in different ways, ranging from cognitive-representational (Lyons et al., 2007) to cultural-normative (Kenward et al., 2011) and social-affiliative hypotheses (Meltzoff, 2007; Nielsen, 2006; Over & Carpenter, 2013). For dogs, we favor the two less representational, cognitive-interpretative hypotheses. We doubt that dogs have been causally confused as a consequence of the humans’ intentional demonstration of the two actions and mistook them to be causally relevant (Lyons et al., 2007). Rather than attempting to solve instrumental problems, they may have interpreted the whole test as a social game, like playing fetch. The replication of the shown actions are not mistaken as being irrelevant for achieving an instrumental goal, but as relevant for the social game. Playing games rather than solving instrumental problems may be the default interpretational stance in companion dogs vis-à-vis the human partner.

Using the caregiver as the demonstrator followed the rationale that the affiliative bond between the human demonstrator and the observing dog would facilitate overimitation. The dog–human relationship is asymmetrical, based on cognitive and behavioral similarities between dogs and children (Hare & Tomasello, 2005). More specifically, this relationship between companion dogs and their human caregivers bears a remarkable resemblance to an infant attachment bond: Dogs are dependent on human care, and their behavior seems specifically geared to engage their caregiver’s care-giving system (Archer 1997; Gácsi, Miklósi, Dóka, & Csányi, 2001; Prato-Previde, Custance, Spiezio, & Sabatini, 2003; Prato-Previde & Valsecchi, 2014; Topál, Miklósi, Csányi, & Dóka, 1998). There is ample evidence that this affiliative bond changes the way the dog explores objects and performs in cognitive tasks (Horn et al., 2012; Horn et al., 2013). These studies have also shown that dogs pay more attention to their owners’ actions than to the actions of other familiar humans (Horn et al., 2013), indicating that their attention is enhanced by affiliation. Such affiliative relationships have very likely not existed in the great apes that have been tested for imitation or overimitation (Clay & Tennie, 2017; Horner & Whiten, 2005). Although, from a purely instrumental point of view, the behavior of the dogs seems maladaptive on first sight, it is beneficial for strengthening the bond between them and their caregiver. This account follows the same logic as theories of human overimitation that emphasize social-motivational aspects. They put special emphasis on imitators’ motives to affiliate with the model by reproducing his or her actions very precisely (Nielsen & Blank, 2011; Over & Carpenter, 2013). Still, we cannot make a strong claim here, as this would require a control experiment with a human demonstrator that is unknown to the dogs. In this regard, it is interesting that dogs learn equally well from a stranger and their owner in a detour task demonstration (Pongrácz et al., 2001).

A further reason for seeing overimitation in dogs but not great apes is in terms of the special, educational context. Companion dogs are often trained by their caregivers or other humans in many different ways and for many different reasons (Rooney & Cowan, 2011). In the human household, such training aims at teaching the dog what to do and what not to do. This often includes causally opaque actions, such as retrieving the newspaper or laying in a dog bed but not the owner's bed. Furthermore, many dogs undergo agility training, obedience training, and other forms of special-purpose trainings in which they also learn from humans causally opaque action sequences. These training regimes have a strong normative component (specifying what one ought to do) and likely increase obedience and decrease behavior problems in dogs (Clark & Boyer, 1993). The overimitating dogs in our study may have perceived the test situation as similar to training situations in the human household or dog training camps.

Support for this account, in terms of interpreting the demonstrated actions as social conventions (or norms) that should be copied, comes from the comparison between dogs that had received considerable amounts of training and those that had not. Overall, trained dogs performed better—that is, they were more likely to show the analyzed behavior. In contrast, the great apes tested in overimitation experiments and many other observational learning experiments had no such experience of learning from humans but rather had experience in solving instrumental problems. It is reasonable to assume that they focused on receiving food and therefore tried to open the box in the most efficient and, for them, the most natural way. In other words, they emulated the causally relevant actions and ignored the causally irrelevant actions (Clay & Tennie, 2017; Horner & Whiten, 2005).

In conclusion, we provide evidence that dogs spontaneously copy actions demonstrated ostensively by their human caregiver, even if these actions are spatially distant and causally disconnected from a second, immediately following action that has an obvious function (making food available). Although at a group level they showed only modest levels of copying, with only a few instances of faithful copying (true imitation), half of the subjects that observed the irrelevant dot-touching action approached the dots and touched at least one. This number of overimitative actions compares well with the dogs in Johnston et al. (2017) and creates a stark contrast to great apes, of which not a single individual ever copied the visibly causally irrelevant action. Rather than differences in capacity, we assume differences in motivation relating to the strong affiliative and perhaps also normative drivers of social learning from humans in dogs but not in great apes (which may, of course, show the same tendencies toward conspecifics). Similar to children, dog’s learning from and copying their caregiver seems to be a profoundly social process. Thus, copying visibly causally irrelevant actions can no longer be seen as a uniquely human action but one shared with their canine companions.

## Electronic supplementary material

ESM 1 (DOCX 42.7 KB)
