# Supplementary material for: Would dogs copy irrelevant actions from their human caregiver?
Source: Learn Behav. 2018 Jul 6;46(4):387–97. doi: 10.3758/s13420-018-0336-z (PMC6276069; doi:10.3758/s13420-018-0336-z)
Supplement: Supplementary file 1 — (DOCX 42.7 KB) [file 13420_2018_336_MOESM1_ESM.docx]

Supplementary Information

**Table S1**: *Individual characteristics of dogs that participated in the study, ordered by group and age (in months). TA target training, TO touchscreen training, EY eye-tracking training*

| **No.** | **Group** | **Age** | **Sex** | **Breed** | **TA** | **TO** | **EY** |
| --- | --- | --- | --- | --- | --- | --- | --- |
| 1 | IRR+REL | 14 | M | Eurasian | no | no | no |
| 2 | IRR+REL | 18 | F | Mixed Breed | no | no | no |
| 3 | IRR+REL | 23 | M | American Staffordshire Terrier | no | no | no |
| 4 | IRR+REL | 31 | M | Belgian Shepherd Dog (Malinois) | no | no | no |
| 5 | IRR+REL | 34 | F | Mixed Breed | no | no | no |
| 6 | IRR+REL | 40 | M | Border Collie | yes | yes | yes |
| 7 | IRR+REL | 41 | M | Mixed Breed | yes | no | no |
| 8 | IRR+REL | 61 | M | Border Collie | no | no | yes |
| 9 | IRR+REL | 65 | F | Bernese Mountain Dog | yes | no | no |
| 10 | IRR+REL | 74 | M | Australian Shepherd | yes | no | no |
| 11 | IRR+REL | 77 | F | Jack Russel Terrier | yes | yes | no |
| 12 | IRR+REL | 98 | F | Hungarian Short-Haired Pointer | yes | no | no |
| 13 | IRR+REL | 103 | F | Border Collie | yes | yes | yes |
| 14 | IRR+REL | 118 | F | Flat Coated Retriever | yes | no | no |
| 15 | IRR+REL | 123 | F | Mixed Breed | yes | no | no |
| 16 | REL+IRR | 17 | M | Pyrenean Sheepdog | yes | yes | no |
| 17 | REL+IRR | 22 | F | Australian Shepherd | no | no | no |
| 18 | REL+IRR | 30 | M | Australian Shepherd | yes | no | no |
| 19 | REL+IRR | 41 | M | Alaskan Husky Mix | yes | no | no |
| 20 | REL+IRR | 42 | F | Labrador Retriever | no | no | no |
| 21 | REL+IRR | 52 | F | Australian Shepherd | no | no | no |
| 22 | REL+IRR | 53 | M | Australian Shepherd | no | no | no |
| 23 | REL+IRR | 65 | F | Shepherd Mix | no | no | no |
| 24 | REL+IRR | 88 | M | Miniature Australian Shepherd | yes | no | no |
| 25 | REL+IRR | 90 | F | Pinscher/Whippetmix | no | yes | no |
| 26 | REL+IRR | 94 | M | Mixed Breed | yes | yes | yes |
| 27 | REL+IRR | 98 | M | German Shepherd | no | no | no |
| 28 | REL+IRR | 105 | F | Labrador Retriever | no | no | no |
| 29 | REL+IRR | 115 | M | Pyrenean Sheepdog | yes | yes | no |
| 30 | REL+IRR | 122 | F | Mixed Breed | no | no | no |
| 31 | IRR | 16 | F | Long Haired Collie | no | no | no |
| 32 | IRR | 34 | F | Australian Shepherd | yes | no | no |
| 33 | IRR | 35 | M | Smooth Collie | no | no | no |
| 34 | IRR | 37 | F | Mixed Breed | no | no | no |
| 35 | IRR | 46 | F | German Shepherd | no | no | no |
| 36 | IRR | 53 | F | Elo | yes | no | no |
| 37 | IRR | 55 | M | Border Collie | yes | no | no |
| 38 | IRR | 61 | F | Australian Shepherd | no | no | no |
| 39 | IRR | 65 | M | Beagle | no | no | no |
| 40 | IRR | 75 | M | Jack Russel Terrier | no | no | no |
| 41 | IRR | 79 | M | Mixed Breed | no | no | no |
| 42 | IRR | 90 | F | Hungarian Short-Haired Pointer | yes | no | no |
| 43 | IRR | 102 | M | Jack Russel Terrier | no | no | no |
| 44 | IRR | 105 | M | Border Collie | yes | yes | no |
| 45 | IRR | 139 | F | Labrador Retriever | yes | no | no |
| 46 | REL | 13 | F | Border Collie | no | no | no |
| 47 | REL | 18 | F | Border Collie | no | no | no |
| 48 | REL | 24 | M | Mixed Breed | no | no | no |
| 49 | REL | 25 | M | Labrador Retriever | no | no | no |
| 50 | REL | 30 | M | Golden Retriever | yes | no | no |
| 51 | REL | 46 | M | Czechoslovakian Wolfdog | no | no | no |
| 52 | REL | 58 | F | Border Collie | no | no | no |
| 53 | REL | 73 | F | Mixed Breed | no | no | no |
| 54 | REL | 85 | F | Mixed Breed | no | no | no |
| 55 | REL | 86 | M | Border Collie | yes | no | yes |
| 56 | REL | 95 | F | Rhodesian Ridgeback | no | no | yes |
| 57 | REL | 102 | F | Border Collie | yes | no | no |
| 58 | REL | 102 | M | Rhodesian Ridgeback | no | no | yes |
| 59 | REL | 107 | M | Bearded Collie | no | no | no |
| 60 | REL | 125 | F | Terrier Mix | yes | yes | no |

**Movie S1:** Attention test, Trials 3, 4 and 5. Footage shows the experimenter hiding a piece of sausage in a cup and then the subject approaching the cups and making a choice by touching one cup. The video shows Trial 3 in which the middle cup was baited like in the first two trials, then Trial 4 in which the left cup was baited (testing the A-not-B performance), and then the Trial 5 in which the right cup was baited. The dog, a 10 yrs old female mongrel, performed correctly in Trials 3 and 5, but failed in Trial 4, committing the A-not-B error.

**Movie S2**: Imitation test, group IRR+REL, human demonstration: Footage shows the owner of the dog demonstrating first the irrelevant and then the relevant action. The movie starts 5 min after the end of Movie S1. The owner signals the dog to stay in place, then walks to the paper wall and, after kneeing down, touches the blue dot with the nose, then walks rightwards and, after kneeing down again, touches the yellow dot, then walks leftwards to the food box and, after kneeing down pushes the sliding door leftwards with the nose. The owner takes the piece of sausage, shows it briefly to the dog, then movies rightwards to hide the food box with her body and puts the treat invisibly for the dog back into the food box.

**Movie S3**: Imitation test, group IRR+REL, subject's test performance: Footage shows the behavior of the dog when being released by the experimenter. The movie starts only 5 s after the end of Movie S2. The subject moves to the paper wall and – after sniffing on the floor – touches the blue dots with the nose (8 s after the start), immediately afterwards (<1 s) touches the yellow dot, then walks to the food box and after another 5 s touches the sliding door, sniffing there and pushes it leftwards to get the treat.
